# Maternal and Fetal Outcomes of Acute Leukemia in Pregnancy: A Retrospective Study of 52 Patients

**DOI:** 10.3389/fonc.2021.803994

**Published:** 2021-12-14

**Authors:** Peng Wang, Zhen Yang, Meng Shan, Shenqi Lu, Luwei Zhang, Shijia Li, Shuhong Hu, Hong Tian, Yang Xu, Depei Wu

**Affiliations:** ^1^ Jiangsu Institute of Hematology, National Clinical Research Center for Hematologic Diseases, The First Affiliated Hospital of Soochow University, Suzhou, China; ^2^ Institute of Blood and Marrow Transplantation, Collaborative Innovation Center of Hematology, Soochow University, Suzhou, China; ^3^ Key Laboratory of Thrombosis and Hemostasis of Ministry of Health, Suzhou, China

**Keywords:** acute leukemia, pregnancy, maternal, fetal, outcome

## Abstract

Acute leukemia during pregnancy (P-AL) is a rare disease with limited data regarding the management and outcomes of mothers and fetuses. We retrospectively analyzed the characteristics, pregnancy outcomes and maternal and neonatal prognoses of 52 patients with P-AL collected from January 2013 to December 2020 in our center. Seventeen (32.7%) patients received chemotherapy during pregnancy (exposed cohort), while 35 (67.3%) received chemotherapy after abortion/delivery (nonexposed cohort). Twenty-six (50.0%) pregnancies ended with abortion, and 26 (50.0%) babies were born through spontaneous delivery or cesarean section. Seven infants (26.9%) were born in the exposed cohort, while 19 infants (73.1%) were born in the nonexposed cohort. Fetuses in the exposed cohort had lower gestational ages (P=0.030) and birth weights (P=0.049). Considering the safety of the fetus, seven patients in the exposed cohort received low-dose chemotherapy, one patient received all-trans retinoic acid (ATRA) and one patient only received corticosteroids as induction therapy. Patients received low-dose chemotherapy as induction therapy had a lower complete remission (CR) rate (P=0.041), and more patients in this group received HSCT (P=0.010) than patients received intensive chemotherapy. Patients who delayed chemotherapy in the nonexposed cohort experienced a trend toward a higher mortality rate than patients who received timely chemotherapy (P=0.191). The CR (P = 0.488), OS (P=0.655), and DFS (P=0.453) were similar between the exposed and nonexposed cohorts. Overall, the 4-year overall survival (OS) and disease-free survival (DFS) rates were estimated at 49.1% and 57.8%, respectively. All newborns were living, without deformities, or developmental and intellectual disabilities. Our study indicated that P-AL patients in the first trimester might tend to receive chemotherapy after abortion. Both the status of disease and patients’ willingness should be taken into consideration when clinicians were planning treatment strategies in the second or third trimester. Low-dose or delayed chemotherapy might decrease the efficacy of induction therapy and survival rate of patients, but HSCT could improve the prognosis.

## Introduction

Acute leukemia (AL) is relatively rare in pregnant patients, with an estimated incidence of 1/100,000 to 1/75,000 (1, 2). Moreover, because the average age of females at the time of pregnancy and the incidence of leukemia have increased in recent years (3, 4), the incidence of acute leukemia during pregnancy (P-AL) has shown an upward trend. However, the diagnosis of P-AL may be delayed because of the nonspecific symptoms and signs of leukemia, such as weakness, fatigue, paleness, and difficulty breathing, that can be attributed to pregnancy (5). AL may increase the risk of complications for pregnant women, affect the growth and development of the fetus, and increase the mortality of pregnant women and fetuses. Therefore, it is often recommended to undergo chemotherapy in P-AL, but the cytotoxicity of chemotherapy drugs can easily cause fetal malformations (6, 7). The management of P-AL is always a great challenge in the clinical setting. In addition, the ethical dilemma caused by this situation hinders the progress of clinical trials. The available literature regarding P-AL is composed mainly of small retrospective studies and case reports.

Apart from the challenging therapeutic dilemma for mothers, the risk of fetal malformations and development caused by exposure to chemotherapy *in utero* has aroused our attention. Some studies have reported that fetuses exposed to chemotherapy during the first trimester have an increased incidence of fetal malformations and abortion rate, while cytotoxic agents can be safely administered during the second and third trimesters (6). However, Avilés A and Neri N concluded that chemotherapy could be administered safely at full doses during pregnancy (including in the first trimester) when the possibility of a cure was reasonable (8). The impact of cytotoxic drugs on fetuses exposed during pregnancy remains controversial.

To date, data regarding the clinical characteristics, therapeutic regimen, adverse events, and outcomes of patients with P-AL are limited. In particular, reports investigating fetal characteristics and prognosis are rare. In this study, we retrospectively analyzed eight years of data regarding clinical characteristics, treatment management, and maternal and fetal outcomes in P-AL.

## Materials and Methods

### Patients

In this retrospective analysis, information was collected from the First Affiliated Hospital of Soochow University on patients with P-AL who were diagnosed between January 2013 and December 2020. The diagnosis and classification of all AL patients conformed to the WHO (2016) standards (9), which recommend that bone marrow samples be examined for morphology, immunophenotype, cytogenetics, and molecular biology. Like all other biopsies performed under local anesthesia, a bone marrow biopsy can be safely performed during pregnancy without harming the fetus. According to the Declaration of Helsinki, the Ethics Committee of the First Affiliated Hospital of Soochow University approved our study.

The inclusion criteria were as follows: 1) patients aged from 18 to 40 years old; 2) patients with AL diagnosed during pregnancy; and 3) patients with complete clinical data and follow-up information. The exclusion criteria were as follows: 1) Patients with pregnancies that occurred during or after treatment for AL; and 2) patients with a history of hematologic disease.

The gestational period was divided into the following three trimesters according to the weeks of pregnancy: the first trimester from weeks 0 to 12, the second trimester from weeks 13 to 27, and the third trimester from weeks 28 to 41. Obstetricians who were experienced with complicated pregnancies followed all patients throughout their pregnancies. Treatment strategies were planned and carried out taking into consideration each family’s decisions and the status of disease.

Newborns were examined and taken care of by a professional pediatric team immediately after birth, and a physical examination was performed every six months in the first year after birth, and then once a year. We monitored the status of children by gathering information about their development from their parents through telephone questionnaires.

### Treatment Protocols

The induction therapy for acute myeloid leukemia (AML) included 1) cytarabine combined with anthracycline (idarubicin, daunorubicin) and 2) the CAG regimen or CAG regimen combined with azacitidine/decitabine. The treatment for acute promyelocytic leukemia (APL) included 1) single all-trans retinoic acid (ATRA), 2) ATRA plus arsenic trioxide (ATO), 3) ATRA combined with ATO and mitoxantrone, and 4) ATRA plus idarubicin. The induction schemes for acute lymphoblastic leukemia (ALL) included: 1) vincristine plus prednisone (Pred), 2) idarubicin combined with Pred, and 3) dexamethasone (Dex) for Ph-negative; and 1) imatinib plus Dex/Pred, and 2) idarubicin for Ph-positive. Details of the treatment is shown in **Supplemental Table 1**.

### Supportive Measures

Hematopoietic stimulation and blood transfusion support therapy were provided for patients with neutrophil (Ne) levels < 0.5x10^9^/L, hemoglobin (Hb) levels < 60 g/L and platelet (PLT) levels < 20x10^9^/L after chemotherapy. Patients with active coagulopathy were managed with PLT, fresh frozen plasma, fibrinogen, or prothrombin complex transfusions. Cytoreduction therapy with hydroxyurea (HU) or Dex was used for hyperleukocytosis. The rational use of antibiotics was provided for patients with infection.

### Hematopoietic Reconstruction Standards

In the absence of hematopoietic stimulation and blood component transfusion, the Ne count of the patients was not lower than 0.5 x 10^9^/L for more than 3 days, and PLT counts was not lower than 20 x 10^9^/L for more than 7 days, which were the standards for the reconstruction of granulocytes and megakaryocytes, respectively.

### Clinical Definitions

Investigator-assessed adverse events (AEs) were summarized according to the National Cancer Institute Common Terminology Criteria for Adverse Events (CTCAE) Version 4.0. Efficacy was assessed as complete remission (CR), partial remission (PR), no remission (NR), overall survival (OS), and disease-free survival (DFS). At the cutoff date of June 1, 2021, OS was defined as the duration from diagnosis to death or the last follow-up for all patients. DFS was defined as the duration from the first date of CR to the date of disease progression or death from any cause. The Ages and Stages Questionnaire 3rd edition (ASQ III) (10) and Bayley Scales of Infant and Toddler Development 3rd edition (BSIDIII) (11, 12) were used to conduct a preliminary assessment of the growth and development of children at different ages.

### Statistical Analysis

The Mann–Whitney U test (continuous variables) and the chi-square test or Fisher’s exact test (categorical variables) were used to estimate significant differences between the groups. OS and DFS curves were estimated by GraphPad Prism 8; survival curves were compared using the log-rank test. P values <0.05 were considered statistically significant.

## Results

### Clinical Characteristics of 52 Patients With P-AL

Among all patients, the median age was 28 years (range, 18-39 years), with 14 (26.9%, 14/52) patients more than 30 years old. Fifteen (28.8%, 15/52) patients were primiparous, and 37 (71.2%, 37/52) were multiparous. The median gestational age of diagnosis was 23 weeks+ 5 days (range, 2 weeks+5 days - 41 weeks). Twelve (23.1%, 12/52) patients were diagnosed with AL in the first trimester, 21 (40.4%, 21/52) were diagnosed in the second trimester, and 19 (36.5%, 19/52) were diagnosed in the third trimester. The median levels of white blood cells, Hb, PLT and bone marrow blasts at diagnosis were 13.63 x 10^9^/L (range, 0.88 - 372.16 x10^9^/L), 87 g/L (range, 40 - 126 g/L), 42x10^9^/L (range, 4 - 267 x 10^9^/L) and 77.6% (range, 22-97%), respectively. A total of 28 patients were diagnosed with AML (no-APL), and the remaining patients were diagnosed as follows: 16 with ALL, six with APL, and two with MPAL. Ten (19.2%, 10/28) patients with AML (no-APL) were classified as favorable risk, 14 (26.9%, 14/28) were classified as intermediate risk, and four (7.7%, 4/28) were classified as poor risk. Sixteen patients with ALL (100%, 16/16) were classified as high risk. Next-generation sequencing was performed on the 52 patients to detect 172 leukemia-related gene mutations. In total, 31 (59.6%, 31/52) out of the 52 patients had at least one mutation, and the median number of mutations per patient was two (range, 1-8) (**Supplemental Figure 1**). The median time interval between the diagnosis and the onset of chemotherapy was eight days (range, 1-190 days), with six (11.5%, 6/52) cases exceeding 30 days. Details of the patients and disease characteristics, along with pregnancy outcomes, are provided in **Table 1**.

**Table 1:** Characteristics of the mothers at diagnosis in the exposed and nonexposed cohorts.

	Treatment during pregnancy (17)	Treatment after delivery/abortion (35)	Total (52)	P
**Age, weeks (range)**	..	..	..	..
Median Age	28 (21-38)	28 (18-39)	28 (18-39)	0.776
Median gestational age	15+5 (2+5-31+6)	31+4 (5-41)	23+5 (2 + 5-41)	0.003
**The stage of pregnancy, n (%)**	..	..	..	..
first trimester	6 (35.3)	6 (17.1)	12 (23.1)	0.173
second or third trimester	11 (64..7)	29 (82.9)	40 (76.9)	..
**First diagnosed**	..	..	..	..
WBC (×10^9^/L), median (range)	24.71 (0.88-372.16)	8.4 (1.5-326.19)	13.63 (0.88-372.16)	0.495
Hb (g/L), median (range)	92 (57-123)	87 (40-126)	87 (40-126)	0.349
PLT (×10^9^/L), median (range)	41 (11-115)	43 (4-267)	42 (4-267)	0.578
BM blast (%), median (range)	81 (28-94.5)	61 (22-97)	77.6 (22-97)	0.049
**Type of leukemia, n (%)**	..	..		..
ALL	6 (35.3)	10 (28.6)	16 (30.8)	0.622
AML	6 (35.3)	22 (62..9)	28 (53.8)	0.061
APL	5 (29.4)	1 (2.9)	6 (11.5)	0.011
MPAL	0 (0.0)	2 (5.7)	2 (3.8)	1.000
**ELN risk stratification, n (%) (AML)**	..	..	..	..
Favorable	3 (50.0)	7 (20.0)	10 (19.2)	1.000
Intermediate	3 (50.0)	11 (31.4)	14 (26.9)	0.341
Adverse	0 (0..0)	4 (11.4)	4 (7.7)	0.290
**Previous pregnancy, n (%)**	..	..	..	0.330
yes	14 (82.4)	23 (65.7)	37 (71.2)	..
No	3 (17.6)	12 (34.3)	15 (28.8)	..
**Termination of pregnancy, n (%)**	..	..		0.375
TA or SA	10 (58.8)	16 (45.7)	26 (50..0)	..
CS or SVD	7 (41.2)	19 (54.3)	26 (50.0)	..
**Median time to chemotherapy, days (range)**	3 (1-27)	13 (1-190)	8 (1-190)	0.000

BM, bone marrow; WBC, white blood cell; Hb, hemoglobin; PLT, platelet; SA, spontaneous abortion; TA, therapeutic abortion; CS, cesarean section; SVD, spontaneous vaginal delivery.

Seventeen patients received induction therapy during pregnancy (exposed cohort), while 35 initiated chemotherapy after delivery/abortion (nonexposed cohort). **Table 1** lists the characteristics of the two cohorts. The exposed cohort had a longer gestational age (median, 15 weeks+5 days *vs*. 31 weeks+4 days, P=0.003), more bone marrow blasts (median, 80.8% *vs.* 61%, P=0.049), a shorter interval from diagnosis to chemotherapy [median, 3 (range, 1-27) days *vs.* 13 (range, 1-190) days, P=0.000] and more patients with APL (29.4% *vs.* 2.9%, P=0.011) than the nonexposed cohort. The distributions of other characteristics were similar in both cohorts of patients.

### Pregnancy Outcomes

**Figure 1:**
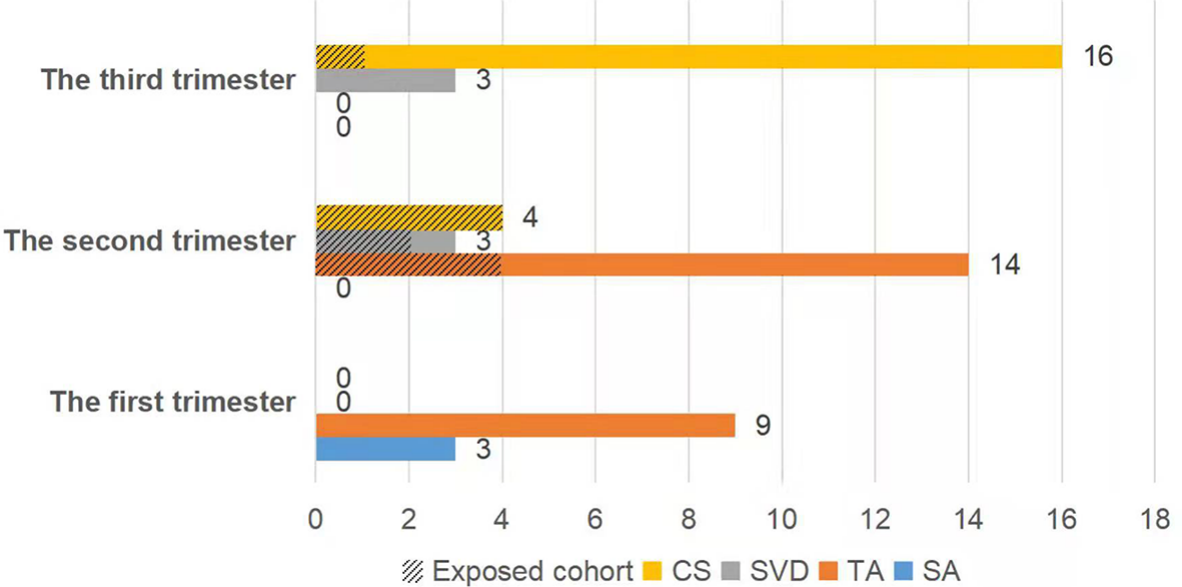
The fetal outcome was dependent on the gestational stage at the time of exposure to chemotherapy. CS, cesarean section; SVD, spontaneous vaginal delivery; TA, therapeutic abortion; SA, spontaneous abortion.

A total of 26 newborns were delivered from 52 women with singleton pregnancies. There were seven neonates (41.2%, 7/17) born in the exposed cohort and 19 newborns (54.3%, 19/35) in the nonexposed cohort (P=0.375). Each patient (100%, 12/12) in the first trimester underwent abortions to terminate the pregnancy. In the second trimester, there were six babies (60.0%, 6/10) born in the exposed cohort and one baby (9.1%, 1/11) born in the nonexposed cohort (P=0.024). And there were one baby (100.0%, 1/1) born in the exposed cohort and 18 babies (100.0%, 18/18) born in the nonexposed cohort during the third trimester (**Figure 1**).

### Maternal Outcomes

**Figure 2:**
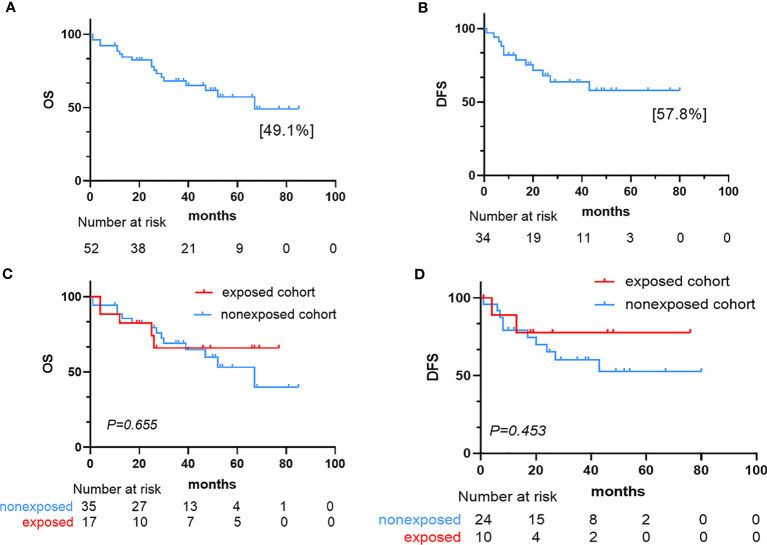
Survival outcome for the 52 AL patients. **(A)** Kaplan–Meier estimate of overall survival (OS) in the 52 patients. **(B)** Kaplan–Meier estimate of disease-free survival (DFS) in the 34 complete responders. **(C)** Kaplan–Meier plots of OS in the exposed and nonexposed cohorts. **(D)** Kaplan–Meier plots of DFS in the exposed and nonexposed cohorts.

After induction therapy, 34 patients (65.4%, 34/52) achieved CR and five (9.6%, 5/52) achieved PR, with an overall response rate ([ORR] = CR+PR) of 75% in the 52 patients. Of the 39 responsive patients, 13 (33.3%, 13/39) received consolidation regimens with maintenance chemotherapy, while 26 (66.7%, 26/39) underwent hematopoietic stem cell transplantation (HSCT). Fifteen (38.5%, 15/39) of 39 responsive patients experienced recurrence, six (40.0%, 6/15) of whom relapsed within six months, and the median time from the first date of CR to relapse was 25.5 months (range, 1-80 months). Thirteen NR patients received reinduction chemotherapy, six (46.2%, 6/13) of whom achieved CR, and seven patients (53.8%, 7/13) maintained NR. Three patients who achieved CR (50.0%, 3/6) received HSCT, and the other three (50.0%, 3/6) received a consolidation regimen combined with maintenance chemotherapy. Five patients (71.4%, 5/7) underwent HSCT with NR; unfortunately, two (40.0%, 2/5) of whom later died from complications of HSCT. The other two NR patients (28.6%, 2/7) continued to receive chemotherapy and died of disease progression (**Table 2**). The median follow-up time of all patients was 33 months. At the last follow-up, we recorded 19 deaths, 13 relapses, and seven cases of nonrelapse mortality (NRM). The most frequent cause of death was relapse. Overall, the 4-year OS and 4-year DFS rates of the 52 patients with P-AL were estimated at 49.1% and 57.8%, respectively (**Figure 2**).

**Table 2:** Maternal and fetal outcomes.

	Trimester in which AL was diagnosed			Total (52)
	First trimester (12)	Second trimester (21)	Third trimester (19)	
**Response to induction, n (%)**	..	..	..	..
CR	7 (58.3)	14 (66.7)	13 (68.4)	34 (65.4)
PR	3 (25)	1 (8.3)	1 (8.3)	5 (9.6)
NR	2 (16.7)	6 (28.6)	5 (26.3)	13 (25)
**Treatment after delivery, n (%)**	..	..	..	..
Chemotherapy	5 (41.7)	7 (33.3)	6 (31.6)	18 (34.6)
Allogeneic transplantation	5 (41.7)	14 (66.7)	13 (68.4)	32 (61.5)
Autologous transplantation	2 (16.7)	0 (0.0)	0 (0.0)	2 (3.8)
**Maternal outcomes, n (%)**	..	..	..	..
Alive	7 (58.3)	13 (61.9)	13 (68.4)	33 (63.5)
Dead	5 (41.7)	8 (38.1)	6 (31.6)	19 (36.5)
**Fetal outcomes, n (%)**	..	..	..	..
Fetal death	12 (100)	14 (66.7)	0 (0.0)	26 (50)
Premature delivery (SVD/CS)	0 (0.0)/0 (0.0)	2 (9.5)/2 (9.5)	0 (0.0)/8 (42.1)	2 (3.8)/10 (19.2)
Term delivery (SVD/CS)	0 (0.0)/0 (0.0)	1 (4.8)/2 (9.5)	3 (15.8)/8 (42.1)	4 (7.7)/10 (19.2)

CR, complete remission; PR, partial remission; NR, non-remission; CS, cesarean section; SVD, spontaneous vaginal delivery.

After induction therapy, the ORR for the exposed cohort was 70.6% (10 patients with CR, 2 patients with PR) compared with 77.1% (24 patients with CR, 3 patients with PR) in the nonexposed cohort (P = 0.735). The CR rate for the exposed cohort was 41.2%, compared with 31.4% in the nonexposed cohort (P = 0.488). The median OS for exposed cohort and nonexposed cohort was not reached and 67 months, respectively. And the median DFS was not reached in two cohorts. The OS and DFS were similar between the two cohorts (OS: P=0.655, DFS: P=0.453). Nineteen patients (36.5%, 19/52) had died at the time of the last follow-up: five (26.3%, 5/19) in the exposed cohort and 14 (73.7%, 14/19) in the nonexposed cohort.

In the exposed cohort, eight patients received intensive chemotherapy, seven patients received low-dose chemotherapy, one patient only received ATRA, and one patient received only corticosteroids as induction therapy. The CR rate after induction of patients received intensive chemotherapy was significantly higher than that of patients with low-dose chemotherapy (87.5% vs. 28.6%, P=0.041). More patients who received low-dose chemotherapy underwent HSCT than those who received intensive chemotherapy (85.7% vs. 12.5% P=0.010), which might be the reason for the similar OS between these patients (OS: P = 0.671) (**Supplemental Figure 2**).

In the nonexposed cohort, six patients delayed chemotherapy, exceeding 30 days. There was no significant difference in the CR rate between the patients who received delayed chemotherapy and timely chemotherapy (83.3% vs. 63.0%, P= 0.651). Four (66.7%, 4/6) of these six patients had died at the time of the last follow-up. The patients who delayed chemotherapy experienced a trend toward a higher mortality rate than patients who received timely chemotherapy (66.7% vs. 34.5%, P= 0.191). However, there were no significant difference in OS and DFS between these patients (OS: P = 0.455, DFS: P=0.687) (**Figure 2**).

### Fetal Outcomes

Among the 52 singleton pregnancies, 26 live newborns were delivered, of which six were delivered by spontaneous vaginal delivery and 20 were delivered by cesarean delivery.

The characteristics and outcomes of live neonates born to mothers with P-AL are shown in **Table 3** and **Supplemental Table 2**. The median gestational age and weight at birth were 37 weeks (range, 29 weeks and 4 days to 41 weeks) and 3000 g (range, 1300-4100 g), respectively. The numbers of preterm infants and term infants were 12 (46.2%) and 14 (53.8%), respectively. There was one (3.8%) infant with ultralow birth weight, 10 infants with low-birth weight (38.5%), 15 (57.7%) infants with normal birth weight, and one (3.8%) infant with high birth weight.

**Table 3:** Characteristics of the living neonates born to mothers with P-AL.

	Entire neonate cohort N=26	Exposed *in utero* to chemotherapy N=7	Nonexposed to chemotherapy N=19	P
**Gestational age in weeks, median (range)**	37 (29 + 4 - 41)	36+4 (29 + 4 - 37 + 4)	38+5 (32 + 2 - 41)	0.030
**Birth weight in g, median (range)**	3000 (1300-4100)	2400 (1300-3350)	3200 (1850-4100)	0.049
**Birth weight category, n (%)**	..	..	..	..
SGA	10 (38.5)	5 (71.4)	5 (26.3)	0.069
AGA	15 (57.7)	2 (28.6)	13 (68.4)	0.095
LGA	1 (3.8)	0 (0.0)	1 (5.3)	1.000
**Delivery timing, n (%)**	..	..	..	0.071
Preterm	12 (46.2)	5 (71.4)	7 (36.8)	..
Term	14 (53.8)	2 (28.6)	12 (63.2)	..
**Complications after birth, n (%)**	..	..	..	0.269
No	25 (96.2)	6 (85.7)	19 (100.0)	..
Yes	1 (3.8)	1 (14.3)	0 (0.0)	..

SGA, small for gestational Age; AGA, appropriate for gestational age; LGA, large for gestational Age; Term: at week 37 or after; Preterm: over week 28 but less than week 37.

Fetuses not exposed to chemotherapy *in utero* had higher gestational ages (median, 38 + 5 weeks vs. 36 + 4 weeks, P = 0.030) and birth weights (median, 3200 vs. 2400, P = 0.049). It seems that fetuses exposed to chemotherapy were likely to experience premature delivery, but this difference did not reach statistical significance (71.4 vs. 36.8, P = 0.071).

A mother who underwent *in vitro* fertilization (IVF) was diagnosed with Ph-positive B-ALL at 23 weeks+5 days of pregnancy and received dexamethasone combined with imatinib chemotherapy during pregnancy. The newborn had slight cerebral hemorrhage after cesarean section at 36 weeks+4 days. Fortunately, the hemorrhage was gradually absorbed during the follow-up.

At the last follow-up, the median age of the 26 newborns was 39.5 (9-81) months. All newborns were alive without deformities, hematological malignancies, and developmental or intellectual disabilities.

## Discussion

The diagnosis of P-AL is a severe event that poses challenges to the pregnant patient, her family, and the medical team. According to limited data, patient with AML during pregnancy (P-AML) accounts for more than two-thirds of P-AL, and most patients were diagnosed during the second and third trimesters (13), To the best of our knowledge, this is the most extensive single-center case series of patients with AL during pregnancy, which includes long-term outcomes of children. Our results regarding the distribution of the disease were similar to previous reports. In this study, most patients had AML (28/52, 53.8%), 30.8% patients (16/52) had ALL, and there were two cases (3.8%) of MPAL and six cases (11.5%) of APL. However, the relationship between pregnancy and gestational age remains unclear. Farhadfar N et al. believed that the P-AL incidence in the first trimester was higher than that in the second and third trimesters. In contrast, Saleh AJ et al. believed that the incidence in the second and third trimesters was higher than that in the first trimester (14, 15), We found that the first, second, and third trimesters of pregnancy accounted for 23.1%, 40.4%, and 36.5%, respectively.

Pregnancy can be considered a unique form of parabiosis in which the mother is tightly joined to the fetus. We were concerned that P-AL might generate a serial effect on the mother and fetus. Although maternal-fetal blood flow is not mixed, regulatory factors may be exchanged that cause both positive and negative effects (16). Obviously, pregnancy is a significant burden for the mother and carries a risk of numerous complications. The evidence obtained thus far was insufficient to determine the relationship between pregnancy and hematological malignancies (1). Whether the changes in female immunity during pregnancy provide opportunities for the occurrence of tumors remains unclear. Bianchi DW et al. reported that maternal immune mediators crossing the placental barrier through various placental pathologies could directly and indirectly alter the uterine environment and influence fetal growth and neurogenesis (17). However, our research showed that the survival rate of mothers was 63.5% at the end of the follow-up, and no malignant hematological disease was found in the children. More research is needed on the interaction between pregnancy and leukemia.

The timing of initiating chemotherapy for P-AL is still controversial. Although limited reports have deemed that chemotherapy can be administered at full doses during any stage of pregnancy without causing fetal malformations (8), most literature has recommended that the termination of pregnancy should be discussed in the first trimester, and chemotherapy treatment during the second or third trimester might not require termination of the pregnancy (6). Greenlund LJ et al. reported that the mortality rate of patients who chose to delay treatment was significantly higher than that of patients who did not chose to delay (18). However, a meta-analysis showed no significant difference between chemotherapy during pregnancy and chemotherapy after pregnancy in terms of remission rates and outcomes (13). Our data showed no significant differences in the CR, OS, and DFS of the patients between the exposed and nonexposed cohorts. Besides, we believed that delayed treatment could be well tolerated in patients with relatively stable conditions. Moreover, Barnes et al. confirmed that chemotherapy could not be delayed in aggressive hematological malignancies due to the fatal risk to the mother and the fetus, secondary to tumor progression (19). Furthermore, our results also showed that receiving low-dose chemotherapy during pregnancy may reduce the efficacy of induction therapy; fortunately, HSCT could compensate for the prognosis of these patients.

There was no consensus on the treatment of P-AL. According to the limited reports, the induction protocols for patients diagnosed with AML during pregnancy include anthracycline and cytarabine. Doxorubicin was the preferred drug during pregnancy since the treatment with doxorubicin was considered relatively safe throughout pregnancy and rare associated with severe congenital malformations (20). While, idarubicin might be associated with higher rates of adverse fetal outcomes because it had an increased placental transfer and affinity to the DNA, and therefore should be avoided during pregnancy (7). The experience with the administration of cytarabine during pregnancy was limited. Animal model data suggested that cytarabine had teratogenic effects (5). In our study, one AML patient who exposed to cytarabine during pregnancy delivered a healthy infant without malformation. And two AML patients who exposed to idarubicin during pregnancy also delivered healthy babies. Patients diagnosed with ALL during pregnancy is rare and most have poor prognosis. The management of ALL during pregnancy is further complicated. There were some reports of patients who exposed to imatinib during pregnancy with no problems for the baby, while, malformations associated with imatinib had also been reported (21). The ALL patient in our study received corticosteroids combined with imatinib during second trimester delivered healthy baby, and had favorable outcome. There was no rational recommendations of treating ALL patients during pregnancy due to the rarely data (5). Further studies are needed on the treatment of the patients with diagnosed ALL during pregnancy. Moreover, APL patients were preferred to receive ATRA based induction chemotherapy during pregnancy in our study. Sanz MA et al. also confirmed that ATRA in the second or third trimester could achieve a high cure rate (22). And ATRA did not appear to increase the risk of fetal complications in the second and third trimester (23). Animal studies suggest that ATO exposures were primarily a risk to the developing fetus. However, the impact of ATO used in pregnant women was unclear (24). There were 3 APL patients used ATO in our study, which did not increase the risk of fetal death, deformity and fetal growth retardation. Although the P-AL patients were rare, our experience in P-AL treatment provided a basis for future research and treatment.

Most published reports have focused on maternal management and the impact of chemotherapy on fetuses exposed *in utero*, and long-term follow-up is rare. Most fetal organs (except the central nervous system and the gonads) are formed in the first trimester, which is the most sensitive stage to chemotherapeutic drug exposure. Studies have shown that cytotoxic chemotherapy drugs could pass through the placental barrier to reach fetal circulation and might increase the risk of spontaneous abortion, fetal death, and severe malformations (25–27). Almost all chemotherapeutics in animal models have been shown to be related to congenital malformations. However, the risk of teratogenicity in humans seems to be lower than that in animals because the doses of chemotherapy used in humans were often lower than the minimum teratogenic doses applied in animals (5). Pentheroudakis G and Pavlidis N reported that the incidence of fetal malformations was 10%-20% when pregnant women received chemotherapy during the first trimester (28), and the abortion rate of patients with P-AL in the first trimester was approximately 40%, which was more than ten times that of normal pregnant women (13). Weisz B et al. and Cardonick E et al. believed that fetal exposure to chemotherapy drugs in the second and third trimesters would not cause malformations; however, it might increase the risk of fetal death, intrauterine growth retardation, neonatal death, premature birth, and low birth weight (7, 29, 30). Similarly, our results showed no congenital malformations regardless of whether fetuses were exposed to chemotherapy *in utero*, but the premature birth rate and low weight risk of the fetuses in the exposed cohort were higher than those in the nonexposed cohort.

We were particularly concerned about whether fetuses exposed to chemotherapy drugs during pregnancy could develop healthily. However, relevant data were quite limited due to the rarity of P-AL and the difficulties in long-term follow-up (5, 30). Peleg and Ben-Ami reviewed the treatment of acute leukemia during pregnancy and did not observe an increase in fetal abnormalities (31). A long-term follow-up study by Aviles A and Neri N showed that the newborns of AL patients who received chemotherapy during pregnancy had normal physical, neurological, and psychological development (8), which was also confirmed in the follow-up data of 111 neonates delivered by patients with different types of malignancies who received chemotherapy during pregnancy (32). Since there was a lack of long-term follow-up data of newborns, we evaluated an eight-year follow-up of children who were exposed to chemotherapy *in utero*. Most newborns were healthy, while only one newborn had hemorrhagic lesions in the brain at birth that were gradually absorbed and did not affect his growth and development. We found that the use of chemotherapeutic agents in the second and third trimesters had no influence on the long-term growth and development, educational performance, or behavior of these children. Moreover, abnormalities were not observed in hematological, renal, hepatic, or cardiac functions.

In this study, P-AL patients in the first trimester might tend to receive chemotherapy after abortion. Both the status of disease and patients’ willingness should be taken into consideration when clinicians were planning treatment strategies. Low-dose or delayed chemotherapy might decrease the efficacy of induction therapy and survival rate of patients, but HSCT could improve the prognosis.

## Data Availability Statement

The original contributions presented in the study are included in the article/**Supplementary Material**. Further inquiries can be directed to the corresponding authors.

## Ethics Statement

The studies involving human participants were reviewed and approved by Ethics Committee of the First Affiliated Hospital of Soochow University. The patients/participants provided their written informed consent to participate in this study.

## Author Contributions

DW, YX, and HT contributed to the conception of the study and manuscript revision. PW, ZY, and MS contributed to collecting and performing the data analysis and preparing the manuscript. SQL, LZ, and SJL helped collect and perform data analysis and prepare the manuscript. SH contributed to the data analysis and manuscript revision. All authors contributed to the article and approved the submitted version.

## Funding

This work was supported in part by grants from the National Natural Science Foundation of China (81730003, 81870120, 81900151, 82070187), the Natural Science Foundation of Jiangsu Province (BK20171205, BK20190176), the Social Development Project of Jiangsu Province (BE2019655), the Jiangsu Province Key R&D Program (BE2019798), the Priority Academic Program Development of Jiangsu Higher Education Institutions (PAPD), and the National Key Research and Development Program (2017ZX09304021, 2017YFA0104500, 2019YFC0840604).

## Conflict of Interest

The authors declare that the research was conducted in the absence of any commercial or financial relationships that could be construed as a potential conflict of interest.

## Publisher’s Note

All claims expressed in this article are solely those of the authors and do not necessarily represent those of their affiliated organizations, or those of the publisher, the editors and the reviewers. Any product that may be evaluated in this article, or claim that may be made by its manufacturer, is not guaranteed or endorsed by the publisher.
