# Supplementary figures and images for: Maternal and Fetal Outcomes of Acute Leukemia in Pregnancy: A Retrospective Study of 52 Patients
Source: Front Oncol. 2021 Dec 14;11:803994. doi: 10.3389/fonc.2021.803994 (PMC8712699; doi:10.3389/fonc.2021.803994)

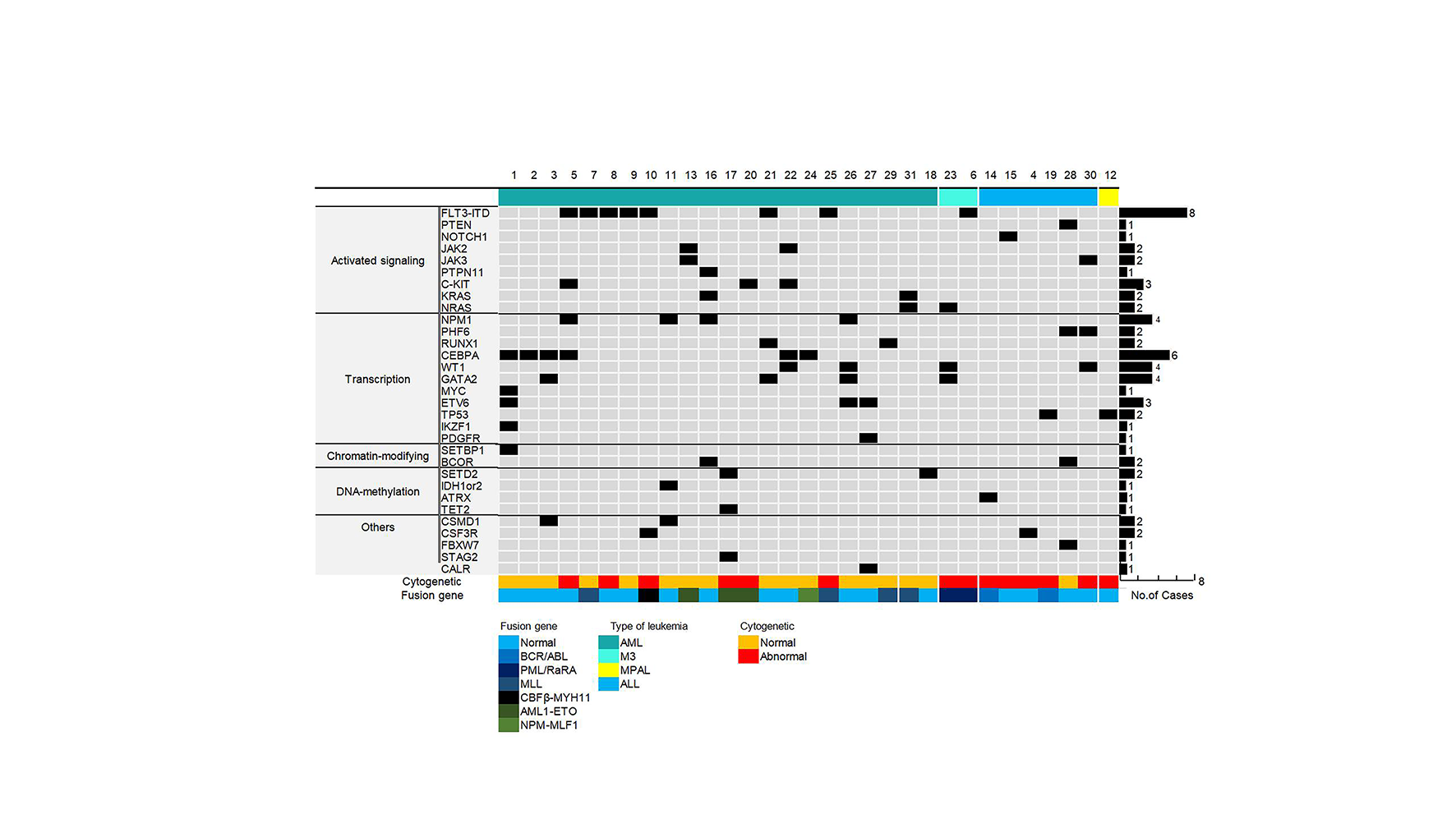

Supplement: Supplementary file 1 [file Image_1.tif]

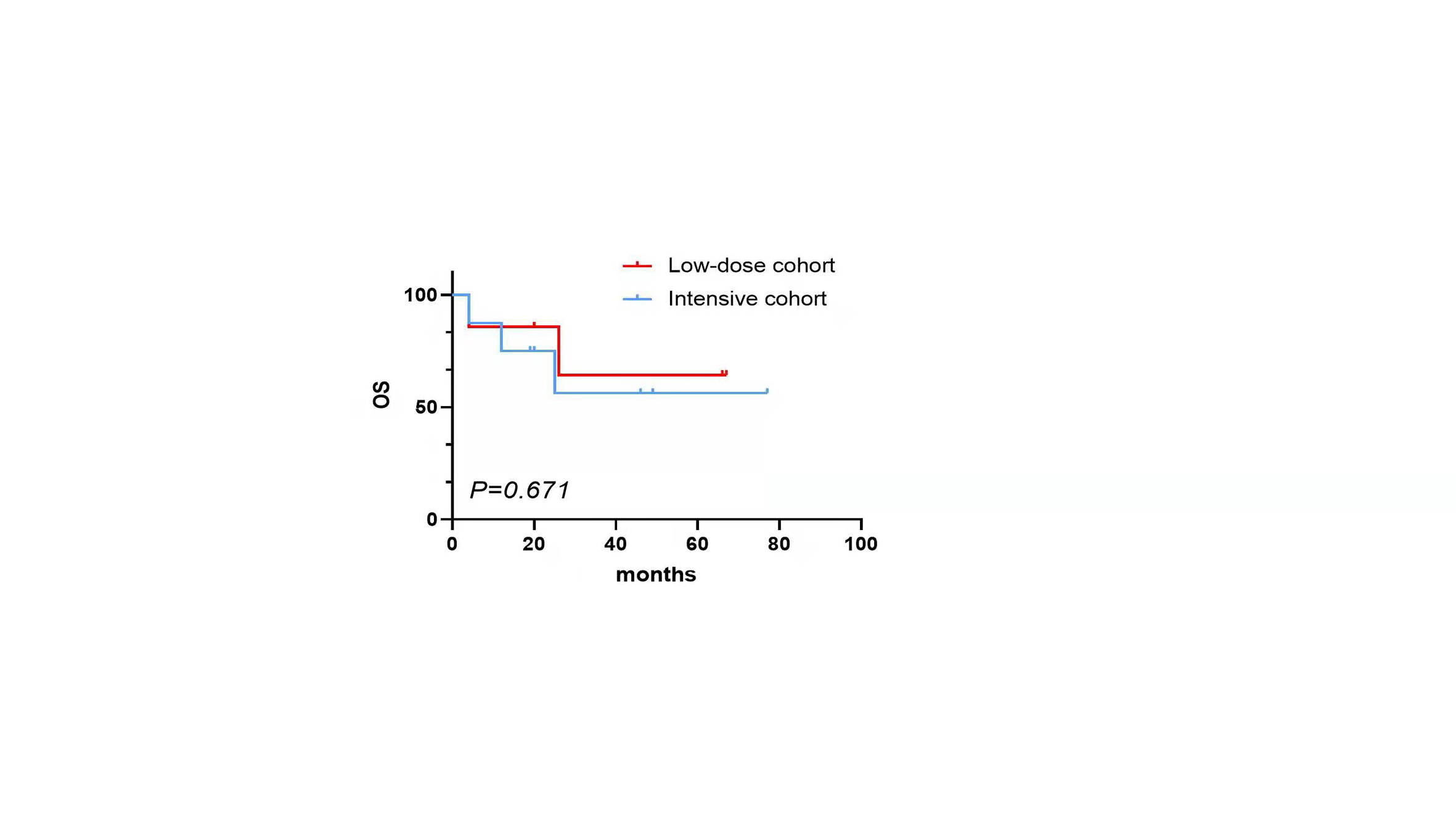

Supplement: Supplementary file 2 [file Image_2.tif]
