# Supplementary material for: Maternal and Fetal Outcomes of Acute Leukemia in Pregnancy: A Retrospective Study of 52 Patients
Source: Front Oncol. 2021 Dec 14;11:803994. doi: 10.3389/fonc.2021.803994 (PMC8712699; doi:10.3389/fonc.2021.803994)
Supplement: Supplementary file 3 [file Table_1.doc]

**Supplemental table 1 Details about induction therapy during pregnancy.**

| **Patient**  **no.** | **Age**  **(yrs)** | **Pregnancy**  **no.** | **AL type** | **Gestational age (wks)**  **At diagnosis at delivery** | **Treatment during pregnancy** | **Pregnancy**  **outcome** | **Fetal outcome** | **Patient**  **outcome** |
| --- | --- | --- | --- | --- | --- | --- | --- | --- |
| P1 | 21 | 2 | AML | 6+3 9+4 | IDA + Ara-C | SA | - | CR |
| P2 | 29 | 1 | AML | 10+2 19+6 | DNR + Ara-C | TA | - | PR |
| P3 | 28 | 2 | AML | 7+2 13+2 | HHT+ Ara-C+G-CSF | SA | - | CR |
| P4 | 28 | 2 | Ph+B-ALL | 2+5 4+1 | HU、IM + Pred | TA | - | CR |
| P5 | 26 | 1 | B-ALL | 7+5 9+4 | HU、Dex | TA | - | NR |
| P6 | 25 | 0 | AML | 6 9 | HU+CTX、DAC+ IDA +Ara-C+G-CSF | TA | - | PR |
| P7 | 28 | 1 | B-ALL | 18 19+6 | VCR + Pred | TA | - | NR |
| P8 | 25 | 1 | T-ALL | 27 33+2 | Pred + IDA | CS | PB | NR |
| P9 | 27 | 1 | Ph+B-ALL | 24+1 36+4 | Dex + IM | SVD | TI | CR |
| P10 | 32 | 2 | Ph+B-ALL | 26+3 32+4 | Pred + IDA | CS | PB | NR |
| P11 | 28 | 3 | APL | 15+5 37+4 | ATRA+ATO+MIT  HU | CS | TI | CR |
| P12 | 24 | 1 | APL | 26+3 29+4 | ATRA+ATO | SVD | PB | CR |
| P13 | 30 | 0 | APL | 20+3 37 | ATRA | CS | PB | CR |
| P14 | 38 | 3 | APL | 13+1 20+4 | ATRA+IDA | TA | - | CR |
| P15 | 34 | 0 | APL | 14 15+6 | ATRA+ATO | TA | - | CR |
| P16 | 28 | 3 | AML | 17+2 23+1 | IDA + Ara-C | TA | - | CR |
| P17 | 23 | 1 | AML | 31 +6 36+5 | THP + Ara-C+G-CSF | CS | PB | NR |

Abbreviations: Ph+: positive for the Philadelphia chromosome ; CR: complete remission; PR: partial remission; NR: non-remission; SA: spontaneous abortion; TA: therapeutic abortion; CS: cesarean section; SVD: spontaneous vaginal delivery; PB: premature birth; TI: term infant; IDA: idarubicin; Ara-C: cytarabine; DNR: daunorubicin; HHT: homoharringtonine; G-CSF: recombinant human granulocyte stimulating factor; HU：hydroxyurea; IM: imatinib; Pred: prednisone; Dex: dexamethasone; CTX: cyclophosphamide; DAC: decitabine; ATRA: all-trans retinoic acid; ATO: Arsenic Trioxide; MIT: mitoxantrone; VCR: vincristine; THP: pirarubicin
