# Supplementary material for: Maternal and Fetal Outcomes of Acute Leukemia in Pregnancy: A Retrospective Study of 52 Patients
Source: Front Oncol. 2021 Dec 14;11:803994. doi: 10.3389/fonc.2021.803994 (PMC8712699; doi:10.3389/fonc.2021.803994)
Supplement: Supplementary file 4 [file Table_2.doc]

**Supplemental table 2** **Characteristics and outcomes of the 26 fetuses.**

| **Neonate**  **no.** | **Weeks of birth** | **Gender** | **Anticancer drugs exposure in utero** | **delivery** | **Apgar score**  **1 min/5 min** | **Birth weights (g)** | **Infant outcome**  **(Age at the survey)** | **Complications in infants** |
| --- | --- | --- | --- | --- | --- | --- | --- | --- |
| N1 | 35 | male | - | CS | - | 2200 | Alive/50months | None |
| N2 | 39+6 | male | - | SVD | - | 3200 | Alive/50months | None |
| N3 | 41 | male | - | SVD | - | 3200 | Alive/40months | None |
| N4 | 39+2 | male | - | CS | - | 4100 | Alive/38months | None |
| N5 | 33+1 | male | - | CS | 8/9 | 2100 | Alive/27months | None |
| N6 | 36+5 | male | THP + Ara-C + G-CSF | CS | - | 2400 | Alive/29months | None |
| N7 | 38 | male | - | SVD | - | 3400 | Alive/31months | None |
| N8 | 36+4 | male | Dex + IM | SVD | 9/10 | 3000 | Alive/9months | encephalorrhagia |
| N9 | 37+4 | male | ATRA、HU、MIT、ATO | CS | 10/10 | 3350 | Alive/43months | None |
| N10 | 37 | male | ATRA | CS | - | 2450 | Alive/23months | None |
| N11 | 29+4 | male | ATRA、ATO | SVD | - | 1300 | Alive/19months | None |
| N12 | 36+6 | male | - | CS | 10/10 | 3450 | Alive/12months | None |
| N13 | 39+3 | male | - | CS |  | 3000 | Alive/66months | None |
| N14 | 38+5 | male | - | CS | 10/10 | 3920 | Alive/56months | None |
| N15 | 32+6 | male | - | CS | 8/9 | 1850 | Alive/21months | None |
| N16 | 38+6 | male | - | CS | 10/10 | 3400 | Alive/10months | None |
| N17 | 38+6 | female | - | CS | - | 3400 | Alive/69months | None |
| N18 | 40+5 | female | - | CS | - | 3100 | Alive/36months | None |
| N19 | 32+2 | female | - | CS | - | 1900 | Alive/29months | None |
| N20 | 33+2 | female | Pred + IDA | CS | 9/9 | 1950 | Alive/64months | None |
| N21 | 32+4 | female | Pred + IDA | CS | 8/10 | 2000 | Alive/60months | None |
| N22 | 36+5 | female | - | CS | 10/10 | 2900 | Alive/57months | None |
| N23 | 35+4 | female | - | CS | 10/10 | 2730 | Alive/52months | None |
| N24 | 41 | male | - | SVD | - | 4000 | Alive/81months | None |
| N25 | 40+6 | male | - | CS | - | 3800 | Alive/58months | None |
| N26 | 37 | female | - | CS | 10/10 | 2350 | Alive/39months | None |

Abbreviations: CS: cesarean section; SVD: spontaneous vaginal delivery; THP: pirarubicin; Ara-C: cytarabine; G-CSF: recombinant human granulocyte stimulating factor; Dex: dexamethasone; IM: imatinib; ATRA: all-trans retinoic acid; HU: hydroxyurea; MIT: mitoxantrone; ATO: Arsenic Trioxide; Pred: prednisone; IDA: idarubicin
